# Research Before Policy: Identifying Gaps in Salmonid Welfare Research That Require Further Study to Inform Evidence-Based Aquaculture Guidelines in Canada

**DOI:** 10.3389/fvets.2021.768558

**Published:** 2022-01-25

**Authors:** Leigh P. Gaffney, J. Michelle Lavery

**Affiliations:** ^1^National Animal Welfare Representative, Code Development Committee (NFACC) for the Code of Practice for the Care and Handling of Farm Animal Care Council (NFACC), Ottawa, ON, Canada; ^2^Department of Biology, University of Victoria, Victoria, BC, Canada; ^3^Scientific Committee (NFACC) for the Code of Practice for the Care and Handling of Farmed Salmonids, National Farm Animal Care Council (NFACC), Ottawa, ON, Canada; ^4^Department of Integrative Biology, University of Guelph, Guelph, ON, Canada

**Keywords:** salmonid, aquaculture, fish welfare, policy, future directions, Canada

## Abstract

Aquaculture is a growing industry worldwide and Canadian finfish culture is dominated by marine salmonid farming. In part due to increasing public and stakeholder concerns around fish welfare protection, the first-ever Canadian Code of Practice for the Care and Handling of Farmed Salmonids was recently completed, following the National Farm Animal Care Council's (NFACC) rigorous Code development process. During this process, both the Scientific (responsible for reviewing existing literature and producing a peer-reviewed report that informs the Code) and Code Development (a diverse group of stakeholders including aquaculture producers, fish transporters, aquaculture veterinarians, animal welfare advocates, food retailers, government, and researchers) Committees identified research gaps in tandem, as they worked through the literature on salmonid physiology, health, husbandry, and welfare. When those lists are combined with the results of a public “top-of-mind” survey conducted by NFACC, they reveal several overlapping areas of scientific, stakeholder, and public concern where scientific evidence is currently lacking: (1) biodensity; (2) health monitoring and management, with a focus on sea lice infection prevention and management; (3) feed quality and management, particularly whether feed restriction or deprivation has consequences for welfare; (4) enclosure design, especially focused on environmental enrichment provision and lighting design; and (5) slaughter and euthanasia. For each of these five research areas, we provide a brief overview of current research on the topic and outline the specific research gaps present. The final section of this review identifies future research avenues that will help address these research gaps, including using existing paradigms developed by terrestrial animal welfare researchers, developing novel methods for assessing fish welfare, and the validation of new salmonid welfare indices. We conclude that there is no dearth of relevant research to be done in the realm of farmed salmonid welfare that can support crucial evidence-based fish welfare policy development.

## Introduction

The number of fish bred, raised, and slaughtered each year for food is on the rise as the human population continues to rapidly increase (1). Due to a decline in capture fisheries worldwide (2, 3), there has been a subsequent expansion of the aquaculture industry to match fish production with increasing consumer demand (4). This trend has led to public interest and concern around aquaculture practices worldwide (5–9) and particularly their impact on fish welfare, which is now a high priority concern for consumers (10, 11) and a policy agenda item (12, 13). However, compared with farmed terrestrial species, fish have not been a priority for welfare researchers for nearly as long (9, 14–16), and thus there exists an urgent need to further our understanding to protect and improve their welfare in aquaculture.

Though elsewhere much of the recent industry expansion has centered around freshwater species, in Canada, the aquaculture industry is dominated by marine salmonid farming, which is valued at ~$1.1 billion per year (17). Accordingly, the first-ever Canadian Code of Practice for the Care and Handling of Farmed Salmonids was recently completed [available at (18)], following the National Farm Animal Care Council's (NFACC) rigorous Code development process [see (19) for details on the development process]. Briefly, the process began with an online survey (reply window: February 26th- March 18th, 2019) asking stakeholders (including those in the farmed finfish industry), key partners, and concerned citizens for their “top of mind” welfare concerns for farmed fish in Canada [see (20) for survey results]. Two committees were then formed: (1) the Scientific Committee, comprised of experts in fish physiology, behavior, health, and welfare, who were tasked with reviewing scientific evidence on priority welfare issues and writing a peer-reviewed report [see (21) for Scientific Committee report]; and (2) the Code Development Committee, who used the Scientific Committee's report to develop the Code's specific requirements and recommendations. Members of the Code Development Committee were a diverse group of stakeholders including aquaculture producers, fish transporters, aquaculture veterinarians, animal welfare advocates, food retailers, government officials, and researchers. Dr. Victoria Braithwaite served as the National Animal Welfare Representative on the Code Development Committee and was an integral contributor to preliminary drafts of the Farmed Salmonids Code of Practice.

## Objective and Identification of Research Gaps

During the NFACC Code development process, both the Scientific and Code Development Committees identified research gaps in tandem, as they worked through the literature on salmonid physiology, health, husbandry, and welfare. When those lists are combined with the results of the public “top-of-mind” survey, they reveal several overlapping areas of concern where scientific evidence is currently lacking (Table 1), to the point where making specific and measurable Code requirements and recommendations was difficult for the Code Development Committee. Thus, herein, our objective is to highlight five of these overlapping welfare-relevant research areas that contain significant knowledge gaps (Table 1): (1) biodensity; (2) health monitoring and management, with a focus on sea lice infection prevention and management; (3) feed quality and management, particularly whether feed restriction or deprivation has consequences for welfare; (4) enclosure design, especially focused on environmental enrichment provision and lighting design; and (5) slaughter and euthanasia.

**Table 1:** Illustration of Research Gaps arising from the “top-of-mind survey” conducted by NFACC, the list of “outstanding issues not addressed in current literature” created by the Scientific Committee and circulated internally, and the list of “research needs” published online by the Code Development Committee (22).

**“Top of mind” survey**	**Scientific Committee**	**Code Development Committee**	**Research gaps**
Top five concerns raised: - **Stocking density^1^** - **Health monitoring and management^2^** - **Humane euthanasia and slaughter^5^** - Water quality - Humane handling Additional concerns raised: - **Feed quality^3^** - **Enclosure design and maintenance^4^** - Behavioral monitoring and management - Emergency preparedness - Transportation	Report chapters with the most “outstanding issues not addressed in current literature” identified by chapter authors: - **Biodensity^1^** (6 issues) - **Sea Lice: Infestation and Treatment^2^** (8 issues) - **Feed Deprivation^3^** (5 issues) - **Lighting^4^** (4 issues) - Stress Indicators (4 issues) - Water Quality Issues in Recirculating Aquaculture Systems (4 issues) - **Ice Slurry Slaughter^5^** (2 issues)	Preliminary “research needs” list identified by the entire Code committee: - **Rearing Units** (5 issues; including topics on biodensity, environmental enrichment, and lighting)^**1,4**^ - **Feeding Management** (2 issues)^**3**^ - **Sea Lice** (5 issues)^**2,4**^ - **Other** (5 issues; including topics on euthanasia and stress)^**3,5**^	Top five overlapping research areas containing significant knowledge-gaps: 1. **Biodensity** 2. **Health monitoring and management (with focus on sea lice)** 3. **Feed quality and management** 4. **Enclosure design (with focus on environmental enrichment and lighting)** 5. **Slaughter and euthanasia**

*Superscript numbers indicate which issues identified by each group were combined to become the research gaps discussed herein*.

For each of these five research areas, we provide a brief overview of current research on the topic and outline the specific gaps present in the current literature, with the final section of this paper identifying future research avenues that will help address these gaps, ideally in advance of future Code revisions. Specific research gaps we report on within each research area were identified by the Scientific and Code Development Committees during numerous meetings over the course of the 3-year Code development process, using both their own extensive reviews of the literature and their collective expertise spanning long research careers in fish physiology and aquaculture [see the Scientific Committee's membership, detailed in (21)] and as aquatic veterinarians and aquaculture professionals (see the Code Development Committee's membership in the Code available at: https://www.nfacc.ca/codes-of-practice/farmed-salmonids). Similar approaches that incorporate multiple perspectives from a variety of stakeholders have been encouraged in the field of animal welfare [e.g., (18, 23, 24)]. Importantly, we do not attempt to provide a comprehensive review of current salmonid welfare research nor a value judgment on what the most pressing future welfare research priorities are. Rather, we are reporting on and extending the work of a unique grouping of aquaculture experts, to highlight future research that is necessary for the continued development of evidence-based salmonid welfare policy in Canada, and thus likely elsewhere as well.

## Definition of Welfare and How It Is Assessed

There exist numerous definitions of “animal welfare” [c.f. (25–27)]. NFACC's current definition includes consideration of affective states, as well as health and biological functioning, and exhibition of both normal and important behaviors. This definition mirrors the “three circles of welfare” approach outlined by Fraser (26), which posits that welfare is comprised of three overlapping concepts (in no particular order): (1) health and biological functioning, (2) affective states, and (3) natural living. Similarly, the Five Freedoms concept, as employed by the OIE (28), includes reference to affective states with words like “comfortable,” “suffering,” “fear and distress,” and “pain.” Though these different concepts have each received criticism [c.f. e.g., (29–32)], a unifying characteristic among them is that the ability to experience pain, suffering, or any other objectionable, negative affective state (i.e., to be capable of sentience) is relevant to welfare. So, following Duncan (25), we take an affective states approach to welfare herein. There still exists some debate around whether fish are capable of sentience [cf. e.g., (33–35)]; however, similar to the Code Development Committee, in this paper we will be taking a precautionary approach that assumes fish are sentient and capable of suffering and experiencing other negative affective states.

The scientific assessment of animal welfare is dependent on validated and standardized measurable parameters known as “welfare indicators.” Welfare indicators can be used to gain insight into an animal's welfare state and can either be direct, animal-based indicators (e.g., weight loss, fin damage, increased gasping at the surface) or indirect, environment-based indicators, centered on the resources and environment the animals are subjected to (e.g., water temperature, oxygen levels) (36–38). Most animal welfare assessment protocols use a combination of both animal and environmental indicators [e.g., (22, 39, 40)] and “operational” welfare indicators are those which are relevant, easy to use, reliable, comparable, suitable for aquaculture and appropriate for specific systems or routines (38). Although a number of validated operational welfare indicators have been developed for salmonids [e.g., (38, 41)], currently there is an ongoing debate and no consensus on the best set of indicators to use [e.g., Salmon Welfare Index Model (SWIM 1.0); the FISHWELL handbook] for assessment of salmonid welfare in aquaculture. The literature reviewed herein uses a variety of operational welfare indicators that we have reported where possible.

## Research Gaps

### Biodensity

Salmonids have a wide range of social behaviors, depending on life-stage [c.f. e.g., for Atlantic salmon: (42, 43)] and species [c.f. e.g., juvenile Arctic charr vs. Atlantic salmon: (42, 44)], so inappropriate biodensities can impact their welfare in captivity. “Biodensity” (often used interchangeably with “stocking density”) is defined as the fish biomass per unit volume of water (usually in units of kg/m^3^). Though biodensity can facilitate useful comparisons, it is important to recognize that fish are rarely distributed consistently throughout a tank or net pen (21) and can instead cluster together or break into smaller groups depending on the species and enclosure conditions. As well, stocking density is constantly changing over time and will increase as fish grow or may decrease following grading or other farming procedures. Considering that fish density can influence water quality depending on flow of water per unit time through the system and that living in water enables fish to move freely in three dimensions (45), the concept of minimum space for fish is thus more complex than for terrestrial animals.

In the context of welfare, biodensity has important implications for managing water quality in net pens, tanks, and recirculating aquaculture systems. But changing the spatial relationship between conspecifics (i.e., altering biodensity independent of water quality considerations) in and of itself can have important implications that change depending on the species and life-stage in question, which makes it challenging to provide blanket guidelines, much less legislation on maximum densities (46). For example, stress response activation increases with increasing biodensity for Atlantic salmon [e.g., highest at 70 kg/m^3^; (47), 125 kg/m^3^; (48)], but increases with decreasing biodensity for Arctic charr [highest at 30 kg/m^3^; (49)]. Aggression follows a similar pattern, with young rainbow trout being most aggressive at high biodensities [e.g., 316 fingerlings/m^3^: (50); > 1000 fingerlings/m^3^: (51)] and young-of-the-year Arctic charr showing the most aggression at low biodensities [44 kg/m^3^; (44)], with adult Atlantic salmon exhibiting increased aggression during feeding (43). Moccia et al. (21) review further examples of how biodensity can impact the health and social behavior of several different salmonid species.

Concerningly, most of the data on optimal biodensities come from experiments conducted in small rearing tanks with relatively small fish (typically parr), due to financial and spatial constraints on research. Thus, findings from experimental manipulations may not be scalable to large production systems, which may use different tank materials or have different tank wall surface to water volume ratios. For example, a typical rearing tank with 1 m diameter and 1 m depth has a ratio of tank wall surface area to water volume of 5:1, while a tank with 5 m diameter and 2 m depth has a ratio of 0.9:1 (21). This might be pertinent when assessing welfare indicators such as fin erosion, a condition in which fins are injured that is hypothesized to be due to abrasion against tank walls and/or conspecific aggression that persists likely due to secondary infection (52). Furthermore, net pens are flexible structures that can change shape in response to tidal and other hydraulic conditions and/or biofouling, which may affect how much living space is available at any given time (53). Finally, a number of biodensity studies are confounded with water quality, such that the results cannot be strictly attributed to the changing number of conspecifics but might be instead a response to deteriorating water quality with increasing density. It is logistically challenging to control water quality in these types of studies, but this can limit how well we can draw clear conclusions on the impact of biodensity independent of other factors.

Beyond the applicability limits of the current research, there exist several crucial gaps in our understanding of how biodensity might impact salmonid welfare. First, we were unable to find studies where salmonid behavioral preferences for different biodensities were tested. Determining what densities different species and life-stages might choose for themselves would be challenging but may provide additional information about which biodensities could optimize salmonid welfare. Second, comparative studies, where species-specific responses to identical experimental parameters are compared, would be of considerable value, especially when trying to extend existing results from one species to many. Third, furthering our understanding of natural salmonid social behavior and how social interactions change with life-stage is important for making biodensity adjustments throughout rearing. As mentioned previously, species-level differences in responses to biodensity can be pronounced, but the salmonid life cycle is also complex, with variation in responses even between life-stages. For example, we know that Atlantic salmon conspecific interactions change a great deal from the parr to adult stages [e.g., (54–58)]. But how much variation is there between life-stages for other salmonids? And what is the relationship between fish size and optimal biodensity? For details of the salmonid life cycle and the dynamic ecology of different life-stages, see Aas et al. (59). Fourth, biodensities are often higher during situations involving acute stress, such as handling and transport. We do not have a strong grasp of what species-specific biodensities could protect welfare in those situations while remaining logistically feasible, nor do we know what biodensities optimize recovery from those acute stressors. Finally, there is evidence that non-optimal biodensities may impact immune parameters and subsequent vulnerability to pathogens [(60–62): reviewed in (53)], but we need further research to elucidate how different biodensities might contribute to pathogen transmission within a given enclosure or system; something that is likely pathogen- and host species-specific as well as multi-factorial.

### Health Monitoring and Management

Disease is a major cause of diminished health and increased mortality in salmon aquaculture (63, 64). Regular monitoring of fish appearance and behavior can help to facilitate early identification of health problems that affect welfare and may be associated with bacterial and viral pathogens, parasites, and/or pollutants [e.g., skin lesions, loss of equilibrium, decreased activity, change in feed intake; (7)]. However, even with regular health monitoring in place, sea lice infestations remain one of the most persistent and highly publicized challenges in salmonid aquaculture.

Sea lice are parasitic copepods (within the family Caligidae) of both wild and farmed marine fish, but the rearing densities and conditions present in salmon aquaculture can exacerbate infection intensities when compared with natural conditions (65, 66). Multiple species of sea lice have been found to infect farmed salmon and sea lice biology, infection, and development are highly dependent on water temperature and salinity [reviewed in (21)]. For example, *Lepeophtheirus salmonis salmonis, Caligus elongatus*, and *C. curtis* represents the greatest concern in the North Atlantic (67, 68), and *L. salmonis oncorhynchii, C. clemensii*, and *L. cuneifer* represent the greatest concern in the Northern Pacific (69, 70). Sea lice feed on the skin, mucus, and blood of their hosts and cause tissue damage (71, 72). In cases of severe infestation, sea lice may also cause significant lesions that lead to increased stress, reduced swimming performance, anemia, reduced growth, and they may even act as a vector for other diseases and pathogens [reviewed in (71); reviewed in (72, 73)]. It is thus essential for aquaculture managers to implement appropriate management and intervention strategies that maintain the welfare of farmed fish and attempt to reduce the impacts of severe sea lice infestations on wild salmonid populations in the area (74, 75).

The primary management approach in all major salmon-producing countries is to regularly monitor and report sea lice densities on salmon in sea pens, with mandatory delousing or other sanctions implemented before levels reach pre-determined limits (21). Sea lice thresholds at which intervention is required (i.e., numbers of sea lice of a particular sex or life stage per fish) are different between and even within countries (21). Currently, however, sea lice thresholds are set for conservation purposes rather than out of concern for captive fish welfare, due to the alleged role of sea lice in the decline of wild salmonid populations as a result of louse spillover infections [e.g., (66, 76–78)]. Although prior work has suggested that sea lice infestations become lethal around 0.12–0.15 lice per cm^2^ of fish (37, 79), the impacts of sea lice are largely dependent on host species and size [e.g., salmon lice are rejected more rapidly by Coho, *Oncorhynchus kisutch*, and pink, *O. gorbuscha*, salmon than by Chinook, *O. tshawytscha*, and chum, *O. keta*, salmon; (69, 71, 72)]. Although, lethal limits are a late-stage indicator of welfare impacts; fish may be negatively affected long before their infection burden induces mortality [e.g., (80)]. There is currently very little research on the sub-lethal effects of sea lice infestations on fish welfare across different salmonid species and life-stages. Research that addresses this gap would help policy makers establish firmer, welfare-based sea lice thresholds for when intervention is required [e.g., (81)].

There also exist a couple of important gaps in our understanding of how to control sea lice infestations. First, lice-infected fish are typically treated by applying chemical treatments in tarpaulin-enclosed net pens (to contain the chemicals), as a bath in well-boats, or by including them in feed (82). Currently, in feed treatments are considered advantageous due to their passive implementation (82), in comparison to bathing treatments which may cause stress and mechanical harm to fish through withholding feed and transfer prior to, and crowding and oxygen deprivation during, bathing (45, 83, 84). However, incorrect dosages of chemical sea lice treatments have been shown to cause mortality post-treatment in salmonids, which typically increases with increasing water temperature [e.g., hydrogen peroxide; (84, 85)]. As well, because some treatments are not completely effective and sea lice are becoming resistant to them (86–89), fish are often treated repeatedly over a 2–3 week period. We do not have a complete understanding of how repeated exposure to chemical therapeutants may impact fish welfare. Second, treatment-resistance has prompted a rapid and recent shift to non-chemical approaches to control infections including the use of altered temperature, salinity, and lighting, physical removal, mechanical barriers, and cleaner fish (21). However, preliminary studies suggest that some of these methods may compromise host salmonid welfare. For example, recent work suggests that temperatures used during thermal delousing treatments (28–34°C) may be noxious to fish (90), can initiate panic reactions [exposure <5 min; (90)], and may cause thermal injury [exposure to 34–38°C for 72–140 s; (91)] and even death [exposure to 34–38°C for >2 min; (91)]. Physical de-lousing systems such as the “Hydrolicer” also require fish to be crowded prior to treatment (85), which may induce an acute stress response. In severe cases, some of these methods can lead to elevated post-treatment mortality in comparison to the use of chemical methods (85). Thus, extensive research is needed to determine the potential impacts of these non-chemical sea lice treatments on fish welfare.

Furthermore, the use of a number of cleaner fish species (e.g., lumpfish and wrasse) that eat sea lice directly off host salmonids are gaining popularity as a biological alternative for infestation control. In the context of salmonid welfare, one of the most important considerations is the role that cleaner fish may play in pathogen transfer to salmonids [e.g., *Tenacibaculum maritimum*; (92); and others reviewed in (93)]. The close mixing of cleaner fish with salmon in net pens creates favorable conditions for the emergence and transfer of diseases, especially considering cleaner fish broodstock are often wild-caught and may pose a biosecurity risk (94, 95). However, the welfare of the cleaner fish themselves is of considerable concern because the biology, ecology, and population dynamics of these species are poorly understood. For example, individuals of some species are territorial (96) and territorial behavior may expose cleaner fish to attacks from the larger captive salmon and thus, exposure to injury and unavoidable chronic stress [e.g., (97–99)]. Reports of poor cleaner fish survival in commercial salmon sea nets [e.g., (99, 100)], with some individual farms observing up to 100% mortality or loss [e.g., (101)], add to this concern. There are also important ethical questions to consider when using cleaner fish. For example, cleaner fish are commonly euthanised after each production cycle when salmonids are slaughtered for harvest (95, 102). This leads to demand for additional, replacement cleaner fish at the beginning of the next salmon production cycle [e.g., (94, 95)] and raises the question: do the ethical implications of this practice outweigh the efficiency of cleaner fish as a sea lice control method? So before introducing these species as a legitimate alternative for sea lice control, we need considerable research at both the basic (e.g., describing cleaner fish ecology, behavior, etc.), applied (e.g., investigating welfare of cleaner fish in sea pens, comparing efficacy of cleaner fish to thermal de-lousing), and philosophical (e.g., is this practice ethical?) levels.

### Feed Quality and Management

The quality of the diet, including feed formulation, affect salmonid health and welfare. For example, feeds with insufficient phosphorus cause potentially painful skeletal deformities [reviewed in (103)]. Popular salmonid feeds usually use fish meal and oil as their primary protein sources, which are limited resources whose harvest can have considerable environmental impact (104). In an effort to improve the sustainability of feeding farmed salmonids, alternative protein sources such as insect meals, poultry by-products, plant-based meals (e.g., soybean, canola, etc.) are being investigated and used (105, 106). However, emerging research suggests that some of these products may have welfare-relevant health impacts. For example, feeding unfermented soybean meal to Atlantic and chinook salmon may cause an intestinal inflammatory response (enteritis) that renders fish more susceptible to diseases like furunculosis (107, 108) but supplementing soybean meal with bacterial meal containing *Methylococcus capsulatus* appears to mitigate enteritis in Atlantic salmon (109). This enteritis also appears to differ in severity between species; unfermented soybean meal does not induce enteritis in pink salmon, and is less severe in Atlantic than in chinook salmon (108). Further research is needed to address species- and life-stage-level differences in how these alternative feed formulations may impact salmonid health and welfare.

Similarly, feed restriction (i.e., feeding a reduced ration) or withdrawal (i.e., not providing any feed) can have welfare consequences that are not well-understood. Feed is withheld in a variety of situations during salmon farming; before acutely stressful procedures like grading, vaccination, etc., it is often considered prudent to empty the gut through short-term feed restriction to maintain water quality during holding, lower hypoxia risk through lowered metabolic rates, and reduce the risk of needle damage during peritoneal injections (6). Prior to slaughter, feed may also be withheld for human food safety and product quality reasons [e.g., (110, 111)]. Further, during rarer events like superchill (112, 113), harmful algae blooms (114), and high temperature events (115), feed withdrawal is often required as it prevents death due to freezing, exposure to algal toxins at the water's surface, or elevated activity in temperatures outside a species' optimal range [reviewed in (21)].

Under the assumption that fish have conscious affective states [as (34, 116), and others claim], the most obvious potential welfare consequence of feed restriction or withdrawal would be hunger, an aversive interoceptive state that can include aspects of pain and frustration and may involve considerable individual variation [e.g., (117)]. However, it is still unknown whether fish experience hunger, both because of doubts surrounding whether they are sentient [cf. e.g., (33, 118, 119)] and because most species (and all salmonids) are ectotherms. Warm-blooded farm animals have consistently high energy demands and therefore require regular meals to avoid hunger and maintain metabolism; however, the feed requirements of fish are dependent on temperature, the principal controlling factor of their metabolic rate (120). Recent research indicates that, when held at optimal temperatures, Atlantic salmon post-smolts can tolerate up to 4 weeks without food with negligible impacts on welfare (121). Some species of fish (including salmonids) also exhibit a natural decrease in appetite to the point of fasting during certain periods of their life cycle [e.g., (122)], so it is possible that hunger is either not as strong a motivator for these fish as it is for mammals, or fish have a physiological mechanism that decreases the aversiveness of hunger during these periods. Work done on transgenic salmon has contributed to our understanding of fish appetite [e.g., (123–125)], but much remains unknown about the endocrinological and neurological mechanisms controlling it and the affective component remains elusive.

Furthermore, welfare consequences may vary depending on the severity and duration of feed restriction or withdrawal, with very long-term situations potentially forcing fish into a stage of starvation requiring protein catabolism to mobilize stored nutrients, wherein vital organ function can be compromised (126). But even less severe feed restriction can have behavioral consequences; the sudden onset of restriction can increase aggression rates and subsequent fin damage severity (127), with these behavioral changes potentially becoming permanent, possibly depending on the life-stage at which feed is restricted [e.g., (128)]. There have been multiple calls for further research on the effect of feed withdrawal of varying lengths on stress physiology, behavior, and welfare (6, 110, 129). Currently, feeding regimes are often based on water temperature and calculations made using known relationships between body size and metabolic rate [for fish: on a log-log scale, body mass and standard metabolic rate are linearly related, with a slope of 0.8: (130), explained in (21)], with the aim of maintaining or increasing body mass. However, this method does not incorporate the numerous other factors that may play a role in how severe the welfare consequences of varying periods of feed restriction or withdrawal are such as water quality, species, life-stage, biodensity, and disease status, among likely many others. In contrast, over-feeding (as a possible result of strong dominance hierarchies, incomplete training of personnel, etc.), though less studied, may have welfare consequences such as fouling of the holding tank or net-pen and/or obesity resulting in possible immunological disorders (131).

### Enclosure Design

A variety of rearing unit types and conditions are used in the farmed salmonid industry, ranging from ponds, sea and lake net pens, and land-based flow-through and recirculating systems. Despite this diversity, aquaculture rearing conditions typically lack complexity, most often being plain, impoverished enclosures containing only water. Deliberately adding resources to the environment with the aim of improving fish welfare by meeting their needs and preferences is often termed “environmental enrichment” (132, 133). Environmental enrichment can take many forms, from physical objects added to the rearing unit that increase structural complexity to sensory, social, nutritional, or even occupational enrichment (133). Providing fish with environmental enrichment that increases the complexity of their rearing units while mimicking their natural environments may be an effective way to offer choice (134) and decrease stress responses. Although enrichment strategies are highly dependent on the natural history of the fish species and their preferences, there are some principles that have been found to hold true for several salmonid species used in research and aquaculture. For example, the use of dark tank backgrounds, tank floor substrate, and shelters, has the potential to reduce aggression and consequent fin damage [Rainbow trout: (135–137); Arctic charr: (138); Coho salmon: (139)] and increase survival [Atlantic salmon: (140, 141)].

For a comprehensive overview of environmental enrichment research for cultured salmonid fishes, see Näslund and Johnsson (133); however to date, environmental enrichment research has been conducted mainly under laboratory conditions in small rearing tanks at relatively low biodensities. While several types of environmental enrichment have been adapted to aquaculture out of necessity (mainly in terms of reproduction success), almost nothing is known about the effects of environmental enrichment on fish welfare at the scale of intensive aquaculture. Furthermore, we do not know what, if any, forms of environmental enrichment are preferred by salmonids at different life-stages, nor what types of enrichment might be important for positive salmonid welfare. There are also some concerns about the application of environmental enrichment that require empirical study: some suggest that enrichment may exacerbate accumulation of food particles and feces [e.g., (142)] or act as a vector for pathogens [e.g., (143)] such that the drawbacks may outweigh the benefits. Accordingly, aquaculture managers are often concerned about effective and safe application of environmental enrichment, especially in a large-scale production context. Much more research is needed to investigate what types of environmental enrichment might be effective and feasible to deploy on-farm.

Lighting is another important aspect of housing design in the farmed salmonid industry. Light has three components: color, intensity, and duration (daylength or photoperiod); all of which can potentially influence animal welfare and can be manipulated by increasing or decreasing the number of lights on the farm, or by changing their strength or type (21). Currently, the manipulation of both photoperiod and light intensity represents key management tools used in salmonid aquaculture. For example, various artificial lighting regimes (e.g., extended or reduced day length or continuous, 24-h lighting) are used to induce smoltification, advance or delay the timing of spawning, manipulate sexual maturation, promote fish growth, and prevent suffocation in the early swim-up stages of the salmonid life cycle (21). Concerningly, there are a number of welfare-relevant health and production issues associated with continuous lighting, including disrupted neurological development, reduced bone strength, poor smolt quality, failed smolting, and failed spawning (144–147). Similarly, sudden changes in light intensity or regime cause fear responses, increased oxygen consumption, injuries, or even suffocation in fish (148–150). So although artificial lighting is readily used and manipulated across the salmonid aquaculture industry, research is needed to investigate these welfare concerns. Furthermore, considering that light intensity influences the spatial distribution of fish within a tank, light intensity may be too low at depth in larger, deeper tanks, which could potentially inhibit feeding, growth, and smoltification (151). We need more information about how light distribution differs with depth in a variety of salmonid housing enclosures and how this impacts fish welfare.

### Slaughter and Euthanasia

Generally, when farmed salmonids reach a certain size, they are slaughtered for human consumption, but it is sometimes also necessary to euthanize fish to prevent them from experiencing excessive pain or suffering (e.g., ill, injured, or diseased fish that do not have a reasonable prospect of improvement or do not respond to treatment). A “humane death” is one that is quick, causes minimal stress and pain, and results in a rapid loss of consciousness followed by death without the ability to regain consciousness (152–154). Under the assumption that fish have conscious affective states, humane approaches to the slaughter and euthanasia of farmed salmonids are expected by both society and the aquaculture industry. Importantly, humane slaughter and euthanasia of fish can only be fully achieved by minimizing stress and injury during, as well as, before the killing procedure itself. Considering procedures such as crowding, loading, and transporting fish from their pens to the place where they will be slaughtered or euthanised (e.g., by use of braille nets, pipes, and/or well boats) has the potential to induce stress and injury in fish [e.g., (155–158)], they must be minimized as much as possible in terms of intensity and duration [e.g., (22, 39, 40, 153)].

Aquaculture slaughter and euthanasia techniques are diverse, and fish species vary in their response to different methods [e.g., sensitivity to oxygen deprivation; (159)]. Unfortunately, some of the current methods are unacceptable under the definition of a “humane death” and have instead been developed with a focus on product quality and ensuring personnel safety (45). For example, immersion in CO_2_ saturated water is sometimes used to kill farmed salmonids; however, it is losing popularity because it has been shown to cause narcosis and loss of brain function [e.g., (160)] over several minutes, during which time the fish exhibit pronounced distress and escape behaviors (161, 162). Thus, considering the negative welfare consequences of these methods, they are being phased out and are only permitted for emergency situations [e.g., CO_2_ may still be used for emergency depopulation events; (22)].

Of the methods presently available, when applied correctly, percussive and electrical stunning appear to be among the more humane methods for salmonid slaughter (163, 164), with electric stunning becoming the preferred method in Canada (21). Considering fish can only be stunned by the use of electricity [i.e., not killed; (162, 165)], electrical stunning must be followed by a kill method that prevents recovery of consciousness in order for it to meet requirements for humane slaughter [e.g., (22, 39, 153, 154)]. However, selection of the most appropriate (i.e., humane) method of slaughter in any situation will depend on the fish species, size, life-stage, number of individuals involved, available means of restraint, and personnel skill level [e.g., (22, 39, 153, 154)]. To date, electrical and percussive stunning methods have been tested on a limited number of fish species at harvestable size, mainly in laboratory conditions [e.g., Atlantic salmon, Common carp, Rainbow trout, Gilthead sea bream, European sea bass; reviewed in (166)], leaving gaps in our understanding of the potential of welfare impacts of these methods in additional fish species, at different life-stages, and in commercial settings. This is concerning because, for example, when the electrical current or voltage is too low, or the application duration too short, electrical stunning can be ineffective at stunning fish and thus, has the potential to cause pain [(154, 166); for a review in fish pain see (167)]. As well, additional considerations need to be taken into account for in-water vs. dry/semi-dry electrical stunning procedures such as the conductivity of the water [e.g., stunning a fish in sea water requires more power than fresh water; (154, 168)] and the orientation of the fish [e.g., incorrect orientation of the fish increases the risk of ineffective stunning; (154, 166)], respectively.

Despite existing research on humane salmonid slaughter and euthanasia, a number of research gaps remain that are hindering our understanding of how these different methods might impact salmonid welfare. First, comparisons between fish and mammalian brains are difficult [due to eversion during embryonic development; see (169)]. Thus, what we know about relationships between mammalian brain regions and their functions cannot be directly applied to fish. It is therefore imperative that we continue to research teleost brain region function in commercially relevant lineages. Second, electroencephalography (EEG) has been used to assess brain electrical activity in fish in a number of laboratory experiments and has been shown to be one of the most reliable methods of assessing consciousness [e.g., (162, 165, 170–173)]. However, in a commercial fish farm setting, registration of EEGs is impossible to perform, instead forcing farmers to rely exclusively on behavioral indicators to evaluate the degree of consciousness in fish [e.g., coordinated swimming and escape behaviors, ability to maintain equilibrium, “eye roll” reflex, and ventilatory reflexes; (170)]. The use of behavioral indicators alone are problematic: for example, some commercially used slaughter methods may only induce sedation and/or paralysis in fish without loss of consciousness [e.g., ineffective electrical stunning, ice slurry slaughter; (163, 166, 170, 174, 175)]. Thus, in order to fully validate the use of behavioral indicators of unconsciousness in the absence of EEGs on farms, more research is needed to investigate additional commercially-relevant fish species and a variety of types of slaughter. Third, we do not fully understand what the actual cause of death is during some of the currently used slaughter and euthanasia techniques. For example, the cause of death during ice slurry slaughter, a method of trout (*O. mykiss*) slaughter used in Canada, is unknown but likely to be asphyxiation from either a lack of gill irrigation or hypoxia [(176); reviewed in (21)]. The chilled water reduces the fishes' activity level but may not render the fish insensible to pain and may thus cause a prolonged period of distress before death (163, 176). Understanding the cause of death can thus be important for assessing welfare impacts, since it can play a role in how long a slaughter method takes to cause death and how potentially painful it may be, especially if another method that causes insensibility (e.g., anesthesia) is not used immediately prior. Thus, methodological studies of how to measure fish brain activity and investigation into the improvement or possible further development of humane slaughter and euthanasia methods would be of use for both fundamental and applied work. Finally and importantly, though electrical and percussive stunning methods are the recommended methods of slaughter at present, this does not preclude the discovery of more humane methods in the future.

## Future Directions

Herein, several gaps in the field of salmonid welfare have been identified, with pertinent questions to guide future research summarized in Table 2. However, further, more in-depth work is required to review the full extent of relevant salmonid welfare research and a complete suite of research gaps, beyond those most relevant to policy development in Canada that we have presented in this review. We strongly suggest that researchers consider performing a scoping review (177) of the literature to provide a complete picture of the state of research and identify a full suite of research deficits. Some valuable reports like this already exist, such as the gap analysis study conducted by the Standing Committee on Agricultural Research [SCAR: (178)], and narrative reviews on various relevant topics by Ashley (6), Overton et al. (85), Macaulay et al. (179), Hvas et al. (180), among others. However, since the aquaculture industry includes a variety of expert stakeholders hailing from different backgrounds, we also recommend borrowing methods from the social sciences [e.g., a systematic review of text and opinion (181), survey-based research (182): Chapter 9], and/or participatory methods [discussed in (24)] to help reveal important anecdotal or experiential understanding from working aquaculture professionals that could inform novel research questions or policy developments [as suggested in relation to the issue of surplus dairy calves, by (24)].

**Table 2:** Examples of outstanding research questions that exist in each Research Gap identified herein, as informed by the Scientific Committee, Code Development Committee, and the authors' perspective as fish welfare researchers.

**Biodensity**	▪ What biodensities are preferred by different salmonid species and life-stages? ▪ How does social behavior change throughout the entire salmonid life cycle? Are these patterns species-specific? ▪ What is the relationship between fish body size and optimal biodensity? ▪ Do patterns and relationships identified in the current biodensity literature scale up to large production systems? ▪ How does biodensity affect salmonid recovery from acute stress? ▪ For different welfare-relevant pathogens, how does biodensity contribute to pathogen transmission?
**Health monitoring and management**	▪ What are the sub-lethal effects of sea lice infestations on salmonid welfare, and at what threshold number of lice per fish do they occur at welfare-compromising levels? ▪ What are the welfare impacts of repeated exposure to chemical therapeutants for managing sea lice infections? ▪ How do alternative sea lice treatment methods (e.g., thermal and physical de-lousing, etc.) impact salmonid welfare? ▪ How does the introduction of cleaner fish species to a given enclosure impact the welfare of captive salmonids? ▪ What are the potential areas of concern for cleaner fish welfare?
**Feed quality and management**	▪ Do fish experience hunger as an aversive affective state? ▪ If hunger is aversive to fish, how motivating is it? ▪ How might the aversiveness of hunger interact with different social dynamics (e.g., dominance hierarchies) to impact welfare? ▪ What protein alternative is best for the welfare of different salmonid species? ▪ What period of feed restriction or withdrawal is appropriate (i.e., does not compromise welfare), and how does it change with different environmental conditions?
**Enclosure design**	▪ What types of if environmental enrichments do farmed salmonids prefer at different life-stages? ▪ What types of environmental enrichment positively impact fish welfare at different life-stages? ▪ What types of environmental enrichment are feasible to deploy on-farm? ▪ What effect does the spectral composition of light have on fish welfare at different life-stages? ▪ What effect do differing photoperiods have on fish welfare? ▪ How is light intensity distributed in differing tank depths and how might this affect fish welfare?
**Slaughter and euthanasia**	▪ What brain region(s) is/are responsible for consciousness in fish? ▪ How do we measure brain function in fish? ▪ At what point does unconsciousness occur during differing slaughter and euthanasia methods? ▪ What is the cause of death in slaughter and euthanasia methods (e.g., ice slurry slaughter and electrical or percussive stunning)? ▪ Are there more humane methods of slaughter and euthanasia than presently available?

With regards to the research questions summarized in Table 2, there are many promising methods that may assist in addressing them, particularly non-lethal physiological indicators of salmonid health such as the quantification of water-borne cortisol [e.g., (183)], fin erosion scoring schemes [e.g., (184)], bioelectrical impedance analysis (185), and hematological indicators of health [reviewed in (186)] and stress [reviewed in (187)]. However, many of these research questions remain unanswered, possibly for several reasons: some of the aforementioned indicators and methods have yet to be fully validated [e.g., (188, 189)], some research questions are yet unanswerable because we lack the necessary tools, and/or we, as fish biologists, have not yet pursued interdisciplinary research to its fullest extent.

The field of animal welfare has been largely focused on terrestrial species but offers many experimental paradigms that can be used to investigate the welfare of aquatic species as well. For example, preference tests commonly used by poultry and cattle welfare researchers [e.g., (190)] have been used to investigate what types of environmental enrichment are most preferred by laboratory zebrafish [e.g., (191)]. These simple preference tests can be extended into investigations of motivation, in which a cost is titrated against access to a resource to determine how valuable it is to an animal [e.g., (192)]. For example, using motivation tests, welfare researchers discovered that farmed mink will pay a high “price” for access to pools for swimming and experience a stress response indistinguishable from that elicited by food deprivation when they are prevented from accessing their favorite resource (193). Further, validated tests of judgment bias, a concept borrowed from human psychology in which one's underlying mood state affects whether neutral stimuli are perceived as potentially rewarding (optimistic) or threatening [pessimistic; e.g., (194)], are gaining popularity for assessing non-human animal mood states [e.g., (195)]. For example, a judgment bias task was recently validated for laboratory mice, wherein mice housed with preferred and welfare-improving environmental enrichment and tumor-bearing nude mice showed optimistic and pessimistic responses, respectively (196). Judgment bias tasks have been attempted for zebrafish [e.g., (197)], but a validated method for salmonids has yet to emerge.

Going forward, a focus on methods development (both building on existing tools and experimental paradigms and creating new ones) would help facilitate the necessary research on salmonid welfare. Of particular interest might be the development of validated judgment bias tasks, ways to assess fish motivation for resources, and other behavioral measures of fish distress, fearfulness, etc. for use on-farm, as well as other non-invasive techniques for investigating fish physiological responses. Considerable work describing salmonid natural ecology exists [e.g., (59, 198, 199), among many others]; however, deepening our understanding of their natural behavior across life-stages (especially during enigmatic at-sea life-stages), would help us further develop and validate behavioral indices of welfare. We may also need to explore how other sensory modalities are affected in production, both as potential welfare implications but also to discover new indices—for example, what sounds can salmon in net pens and land-based enclosures detect and/or produce, and are they relevant to welfare state? How do different enclosure designs affect how salmon use their lateral line, and are there properties of the lateral line that are affected by overall welfare? Longer-term, a non-invasive way to measure brain activity in tanks, and further work on fish brain neuroanatomy and function [e.g., (200, 201)], would help us understand and potentially validate new welfare indicators. Overall, developing a suite of validated, non-lethal welfare indicators that facilitate rapid and reliable assessment of welfare on-farm would be of considerable value. Such a panel of indicators could help us understand welfare at the fish level on-farm, since there is likely to be high individual variation in welfare and stress coping ability within a given group of farmed salmonids [e.g., (202)].

There is obviously no dearth of relevant research to be done in the realm of farmed salmonid welfare. In particular, it is essential to address these and other research gaps to ensure that policy guidelines do not rest solely on assumptions about whether these gaps represent welfare issues or not. Evidence-based policies safeguard welfare in meaningful ways while preventing pointless and potentially damaging impacts on valuable industries. Together with the work done by both the NFACC Scientific and Code Development Committees, we hope that this review serves to guide future studies toward the most pressing and policy-relevant research questions, ideally in advance of future NFACC Code of Practice revisions. But regardless of timelines, it is important that we support and conduct basic and applied research that can address some of the gaps in our understanding of how to safeguard farmed fish welfare, especially considering increasing expressions of concern for fish well-being from farmers and the general public and the continuing expansion of the salmonid aquaculture industry.

## Author Contributions

Both authors listed have made a substantial, direct, and intellectual contribution to the work and approved it for publication.

## Funding

This review was funded by the British Columbia Salmon Restoration and Innovation Fund and the University of Victoria.

## Conflict of Interest

The authors declare that the research was conducted in the absence of any commercial or financial relationships that could be construed as a potential conflict of interest.

## Publisher's Note

All claims expressed in this article are solely those of the authors and do not necessarily represent those of their affiliated organizations, or those of the publisher, the editors and the reviewers. Any product that may be evaluated in this article, or claim that may be made by its manufacturer, is not guaranteed or endorsed by the publisher.

## References

[1] Béné C Barange M Subasinghe R Pinstrup-Andersen P Merino G Hemre GI. Feeding 9 billion by 2050–putting fish back on the menu. Food Security. (2015) 7:261–74. 10.1007/s12571-015-0427-z

[2] Pauly D Villy C Sylvie G Tony JP Sumaila UR Walters CJ. Towards sustainability in world fisheries. Nature. (2002) 418:689–95. 10.1038/nature01017 12167876

[3] OECD. OECD Review of Fisheries 2020. Paris: OECD Publishing (2020). 10.1787/7946bc8a-en

[4] FAO. The State of World Fisheries and Aquaculture - Meeting the Sustainable Development Goals. Food and Agriculture Organization of the United Nations (2018). Available online at: http://www.fao.org/documents/card/en/c/I9540EN/ (accessed August 10, 2021).

[5] Huntingford FA Adams C Braithwaite VA Kadri S Pottinger TG Sandøe P. Current issues in fish welfare. J Fish Biol. (2006) 68:332–72. 10.1111/j.0022-1112.2006.001046.x

[6] Ashley PJ. Fish welfare: current issues in aquaculture. Appl Anim Behav Sci. (2007) 104:199–235. 10.1016/j.applanim.2006.09.001

[7] Martins C Galhardo L Noble C Damsgård B Spedicato MT Zupa W. Behavioural indicators of welfare in farmed fish. Fish Physiol Biochem. (2012) 38:17–41. 10.1007/s10695-011-9518-8 21796377 PMC3276765

[8] Brown C Dorey C. Pain and emotion in fishes–fish welfare implications for fisheries and aquaculture. Anim Stud J. (2019) 8:175–201. 10.14453/asj.v8i2.12

[9] Saraiva JL Arechavala-Lopez P Castanheira MF Volstorf J Studer BH. A global assessment of welfare in farmed fishes: the FishEthoBase. Fishes. (2019) 4:30. 10.3390/fishes4020030

[10] Broom DM. Animal welfare complementing or conflicting with other sustainability issues. Appl Anim Behav Sci. (2019) 219:104829. 10.1016/j.applanim.2019.06.010

[11] Keeling L Tunón H Olmos Antillón G Berg C Jones M Stuardo L. Animal welfare and the United Nations Sustainable development goals. Front Vet Sci. (2019) 6:336. 10.3389/fvets.2019.00336 31649940 PMC6797006

[12] Lu J Bayne K Wang J. Current status of animal welfare and animal rights in China. Alternat Lab Anim. (2013) 41:351–7. 10.1177/026119291304100505 24329743

[13] Lundmark F Berg C Schmid O Behdadi D Röcklinsberg H. Intentions and values in animal welfare legislation and standards. J Agri Environ Ethics. (2014) 27:991–1017. 10.1007/s10806-014-9512-0

[14] Walker M Diez-Leon M Mason G. Animal welfare science: recent publication trends and future research priorities. Int J Consum Stud. (2014) 27:80–100. 10.46867/ijcp.2014.27.01.0334359125

[15] Freire R Nicol C. A bibliometric analysis of past and emergent trends in animal welfare science. Animal Welfare. (2019) 28:465–85. 10.7120/09627286.28.4.465

[16] Kristiansen TS Bracke MB. A Brief Look Into the Origins of Fish Welfare Science. In The Welfare of Fish. Cham: Springer (2020). p. 1–17. 10.1007/978-3-030-41675-1_1

[17] Statistics Canada,. Tables 32-10-0107-01 Aquaculture, Production Value. (2019). Available online at: https://www150.statcan.gc.ca/t1/tbl1/en/tv.action?pid=3210010701 (accessed January 13, 2021).

[18] Fernandes J Blache D Maloney SK Martin GB Venus B Walker FR. Addressing animal welfare through collaborative stakeholder networks. Agriculture. (2019) 9:132. 10.3390/agriculture9060132

[19] NFACC. Development Process for Codes of Practice for the Care and Handling of Farm Animals. National Farm Animal Care Council (2021). Available online at: https://www.nfacc.ca/code-development-process (accessed August 3, 2021).

[20] NFACC. At-a-Glance: Farmed Finfish Survey Results. National Farm Animal Care Council. (2019). Available online at: https://www.nfacc.ca/resources/codes-of-practice/finfish/FinalFarmedFinfishReport17June2019.pdf (accessed August 3, 2021).

[21] Moccia RD Scarfe D Duston J Stevens ED Lavery JM. Code Of Practice For The Care Handling Of Farmed Salmonids: Review Of Scientific Research On Priority Issues. NFACC Scientific Committee Report. (2020). Available online at: https://www.nfacc.ca/pdfs/codes/scientists-committee-reports/farmed%20salmonids_SC%20Report_2020.pdf (accessed August 1, 2021).

[22] NFACC. Code of Practice for the Care and Handling of Farmed Salmonids. National Farm Animal Care Council (2021). Available online at: https://www.nfacc.ca/pdfs/codes/farmed_salmonid_code_of_practice.pdf (accessed November 25, 2021).

[23] Weary DM Ventura BA Von Keyserlingk MAG. Societal views and animal welfare science: understanding why the modified cage may fail and other stories. Animal. (2016) 10:309–17. 10.1017/S1751731115001160 26206166

[24] Bolton SE von Keyserlingk MA. The dispensable surplus dairy calf: is this issue a “wicked problem” and where do we go from here? Front Vet Sci. (2021) 8:347. 10.3389/fvets.2021.660934 33937380 PMC8079806

[25] Duncan IJH. Welfare is to do with what animals feel. J Agri Environ Ethics. (1993) 6:8–14.

[26] Fraser D. Understanding animal welfare. Acta Vet Scand. (2008) 50:S1. 10.1186/1751-0147-50-S1-S1 19049678 PMC4235121

[27] Arlinghaus R Cooke SJ. Recreational fisheries: socioeconomic importance, conservation issues and management challenges. In: B Dickson, J Hutton, WM Adams, editors, Recreational Hunting, Conservation and Rural Livelihoods. Hoboken, NJ: Blackwell Publishing. (2009). p. 39–58. 10.1002/9781444303179.ch3

[28] OIE. Terrestrial Animal Health Code. 29th ed. Paris: OIE; World Organisation for Animal Health (2021). Available online at: https://www.oie.int/en/what-we-do/standards/codes-and-manuals/terrestrial-code-online-access/ (accessed October 12, 2021).

[29] McCulloch SP. A critique of FAWC's five freedoms as a framework for the analysis of animal welfare. J Agri Environ Ethics. (2013) 26:959–75. 10.1007/s10806-012-9434-7

[30] Korte SM Olivier B Koolhaas JM. A new animal welfare concept based on allostasis. Physiol Behav. (2007) 92:422–8. 10.1016/j.physbeh.2006.10.018 17174361

[31] Duncan IJ. Is sentience only a nonessential component of animal welfare? Anim Sentience. (2016) 1:6. 10.51291/2377-7478.1023

[32] Mellor DJ. Updating animal welfare thinking: moving beyond the “Five Freedoms” towards “a Life Worth Living”. Animals. (2016) 6:21. 10.3390/ani6030021 27102171 PMC4810049

[33] Rose JD Arlinghaus R Cooke SJ Diggles BK Sawynok W Stevens ED. Can fish really feel pain? Fish Fisheries. (2014) 15:97–133. 10.1111/faf.12010

[34] Sneddon LU Lopez-Luna J Wolfenden DC Leach MC Valentim AM Steenbergen PJ. Fish sentience denial: muddying the waters. Anim Sentience. (2018) 3:1. 10.51291/2377-7478.1317

[35] Vettese T Franks B Jacquet J. The great fish pain debate. Issues Sci Technol. (2020) 36:49–53.24942105

[36] Duncan IJH. Science-based assessment of animal welfare: farm animals. Revue Scientifique et Technique. (2005) 24:483–92. 10.20506/rst.24.2.1587 16358502

[37] Stien LH Bracke MB Folkedal O Nilsson J Oppedal F Torgersen T. Salmon Welfare Index Model (SWIM 1.0): a semantic model for overall welfare assessment of caged Atlantic salmon: review of the selected welfare indicators and model presentation. Rev Aquaculture. (2013) 5:33–57. 10.1111/j.1753-5131.2012.01083.x

[38] Noble C Gismervik K Iversen MH Kolarevic J Nilsson J Stien LH. Welfare Indicators for Farmed Atlantic Salmon: Tools for Assessing Fish Welfare. (2018). p. 351.

[39] RSPCA. Welfare Standards for Farmed Atlantic Salmon. Royal Society for the Prevention of Cruelty to Animals (2018). Available online at: https://science.rspca.org.uk/sciencegroup/farmanimals/standards/salmon (accessed October 21, 2021).

[40] OIE. Chapter 7.2- Welfare of Farmed Fish During Transport. World Organisation for Animal Health (2021). Available online at: https://www.oie.int/en/what-we-do/standards/codes-and-manuals/aquatic-code-online-access/?id=169andL=1andhtmfile=chapitre_welfare_transport_farm_fish.htm#chapitre_welfare_transport_farm_fish (accessed October 12, 2021).

[41] Jarvis S Ellis MA Turnbull JF Rey Planellas S Wemelsfelder F. Qualitative behavioral assessment in juvenile farmed Atlantic Salmon (*Salmo salar*): potential for on-farm welfare assessment. Front Vet Sci.(2021) 2021:1019. 10.3389/fvets.2021.702783 34557541 PMC8453064

[42] Jones HAC Noble C Damsgard B Pearce GP. Social network analysis of the behavioural interactions that influence the development of fin damage in Atlantic salmon parr (*Salmo salar*) held at different stocking densities. Appl Anim Behav Sci. (2011) 133:117–26. 10.1016/j.applanim.2011.05.005

[43] Adams CE Turnbull JF Bell A Bron JE Huntingford FA. Multiple determinants of welfare in farmed fish: stocking density, disturbance, and aggression in Atlantic salmon (*Salmo salar*). Can J Fisheries Aquatic Sci. (2007) 64:336–44. 10.1139/f07-018

[44] Brown GE Brown JA Srivastava RK. The effect of stocking density on the behaviour of Arctic charr (*Salvelinus alpinus* L.). J Fish Biol. (1992) 41:955–63. 10.1111/j.1095-8649.1992.tb02722.x

[45] Conte FS. Stress and the welfare of cultured fish. Appl Anim Behav Sci. (2004) 86:205–23. 10.1016/j.applanim.2004.02.003

[46] Ellis T North B Scott AP Bromage NR Porter M Gadd D. The relationships between stocking density and welfare in farmed rainbow trout. J Fish Biol. (2002) 61:493–531. 10.1111/j.1095-8649.2002.tb00893.x

[47] Sundh H Finne-Fridell F Ellis T Taranger GL Niklasson L Pettersen EF. Reduced water quality associated with higher stocking density disturbs the intestinal barrier functions of Atlantic salmon (*Salmo salar* L.). Aquaculture. (2019) 512:734356. 10.1016/j.aquaculture.2019.734356

[48] Calabrese S Nilsen TO Kolarevic J Ebbesson LOE Pedrosa C Fivelstad S. Stocking density limits for post-smolt Atlantic salmon (*Salmo salar* L.) with emphasis on production performance and welfare. Aquaculture. (2017) 462:363–70. 10.1016/j.aquaculture.2016.10.041

[49] Sevier A Smith R Benfey T Danzmann R Bernier N Moccia R. Effects of biodensity on the growth, stress physiology, and welfare of Arctic charr (*Salvelinus alpinus*) in freshwater. Comparat Biochem Physiol A. (2019) 231:91–103. 10.1016/j.cbpa.2019.01.021 30690147

[50] Keeley ER. An experimental analysis of territory size in juvenile steelhead trout. Anim Behav. (2000) 59:477–90. 10.1006/anbe.1999.1288 10715169

[51] Cole KS Noakes DLG. Development of early social behaviour of rainbow trout, *Salmo gairdneri* (Pisces, Salmonidae). Behav Processes. (1980) 5:97–112. 10.1016/0376-6357(80)90059-5 24897716

[52] Latremouille DN. Fin erosion in aquaculture and natural environments. Rev Fisheries Sci. (2003) 11:315–35. 10.1080/1064126039025574525469952

[53] Turnbull JF North BP Ellis T Adams CE Bron J MacIntyre CM. Chapter 8: stocking density and the welfare of farmed salmonids. In: EJ Branson, editor, Fish Welfare. Hoboken, NJ: John Wiley and Sons (2008). p. 111–20. 10.1002/9780470697610.ch8

[54] Kadri S Huntingford FA Metcalfe NB Thorpe JE. Social interactions and the distribution of food among one-sea-winter Atlantic salmon (*Salmo salar*) in a sea-cage. Aquaculture. (1996) 139:1–10. 10.1016/0044-8486(95)01163-3

[55] Keeley ER Grant JWA. Allometric and environmental correlates of territory size in Juvenile Atlantic Salmon (*Salmo salar*). Can J Fisheries Aquatic Sci. (1995) 52:186–96. 10.1139/f95-019

[56] Dill PA. Development of behaviour in alevins of Atlantic salmon, *Salmo salar*, and rainbow trout,S. gairdneri. Anim Behav. (1977) 25:116–21. 10.1016/0003-3472(77)90073-2

[57] Keenleyside MH. Skin-diving observations of Atlantic salmon and brook trout in the Miramichi River, New Brunswick. J Fisheries Board Canada. (1962) 19:625–34. 10.1139/f62-042

[58] Kallenberg H. Observations in a stream tank of territoriality and competition in juvenile salmon and trout. Rep Inst Freshw Res. (1958) 39:55–8.

[59] Aas Ø Klemetsen A Einum S Skurdal J. Atlantic Salmon Ecology. Hoboken, NJ: John Wiley and Sons (2010). 10.1002/9781444327755

[60] Mazur CF Tillapaugh D Iwama GK. The effects of feeding level and rearing density on the prevalence of *Renibacterium salmoninarum* in chinook salmon (*Oncorhynchus tshawytscha*). Aquaculture. (1993) 117:141–7. 10.1016/0044-8486(93)90130-Q

[61] LaPatra SE Groff JM Patterson TL Shewmaker WD Casten M Siple J. Preliminary evidence of sturgeon density and other stressors on manifestation of white sturgeon iridovirus disease. J Appl Aquaculture. (1996) 6:51–8. 10.1300/J028v06n03_05

[62] Bebak-Williams J McAllister PE Smith G Boston R. Effect of fish density and number of infectious fish on the survival of rainbow trout fry, *Oncorhynchus mykiss* (Walbaum), during epidemics of infectious pancreatic necrosis. J Fish Dis. (2002) 25:715–26. 10.1046/j.1365-2761.2002.00426.x

[63] Murray AG Peeler EJ. A framework for understanding the potential for emerging diseases in aquaculture. Prev Vet Med. (2005) 67:223–35. 10.1016/j.prevetmed.2004.10.012 15737433

[64] Lafferty KD Harvell CD Conrad JM Friedman CS Kent ML Kuris AM. Infectious diseases affect marine fisheries and aquaculture economics. Ann Rev Mar Sci. (2015) 7:471–96. 10.1146/annurev-marine-010814-015646 25251276

[65] Costello MJ. Ecology of sea lice parasitic on farmed and wild fish. Trends Parasitol. (2006) 22:475–83. 10.1016/j.pt.2006.08.006 16920027

[66] Torrissen O Jones S Asche F Guttormsen A Skilbrei OT Nilsen F. Salmon lice–impact on wild salmonids and salmon aquaculture. J Fish Dis. (2013) 36:171–94. 10.1111/jfd.12061 23311858 PMC3675643

[67] Hogans WE Trudeau DJ. Preliminary studies on the biology of sea lice, *Caligus elongatus, Caligus curtus*, and *Lepeophtheirus salmonis* (Copepoda: Caligidae) parasitic on cage-cultured salmonids in the lower Bay of Fundy. Can Technical Rep Fisheries Aquatic Sci. (1989) 1715:14.

[68] Hemmingsen W MacKenzie K Sagerup K Remen M Bloch-Hansen K Imsland AKD. *Caligus elongatus* and other sea lice of the genus Caligus as parasites of farmed salmonids: a review. Aquaculture. (2020) 522:735160. 10.1016/j.aquaculture.2020.735160

[69] Jones SRM Hargreaves NB. The abundance and distribution of *Lepeophtheirus salmonis* (Copepoda: caligidae) on pink (*Oncorhynchus gorbuscha*) and chum (*O. keta*) salmon in coastal. Br Columbia J Parasitol. (2007) 93:1324–31. 10.1645/GE-1252.1 18314676

[70] Jones S Johnson S. Sea Lice Monitoring and Non-Chemical Measures A: Biology of Sea Lice, Lepeophtheirus salmonis and Caligus spp., in Western and Eastern Canada. DFO Canadian Science Advisory Secretariat Research Document 2014/019 Ottawa, ON: Fisheries and Oceans Canada (2015).

[71] Johnson SC Fast MD. Interactions between sea lice and their hosts. Host-Parasite Interactions. (2004) 7:131–59. 10.4324/9780203487709-7 15446448

[72] Wagner GN Fast MD Johnson SC. Physiology and immunology of *Lepeophtheirus salmonis* infections of salmonids. Trends Parasitol. (2008) 24:176–83. 10.1016/j.pt.2007.12.010 18329341

[73] Fjelldal PG Hansen TJ Karlsen O Wright DW. Effects of laboratory salmon louse infection on Arctic char osmoregulation, growth and survival. Conserv Physiol. (2019) 7:coz072. 10.1093/conphys/coz072 31723431 PMC6839430

[74] Patanasatienkul T Sanchez J Rees EE Krkošek M Jones SR Revie CW. Sea lice infestations on juvenile chum and pink salmon in the Broughton Archipelago, Canada, from 2003 to 2012. Dis Aquat Organ. (2013) 105:149–61. 10.3354/dao02616 23872858

[75] Thorstad EB Todd CD Uglem I Bjørn PA Gargan PG Vollset KW. Effects of salmon lice *Lepeophtheirus salmonis* on wild sea trout *Salmo trutta* a literature review. Aquacult Environ Interactions. (2015) 7:91–113. 10.3354/aei00142

[76] McVicar AH. Management actions in relation to the controversy about salmon lice infections in fish farms as a hazard to wild salmonid populations. Aquac Res. (2004) 35:751–8. 10.1111/j.1365-2109.2004.01097.x

[77] Krkosek M Revie CW Gargan PG Skilbrei OT Finstad B Todd CD. Impact of parasites on salmon recruitment in the Northeast Atlantic Ocean. Proc R Soc B Biol Sci. (2013) 280:20122359. 10.1098/rspb.2012.2359 23135680 PMC3574446

[78] Shephard S Gargan P. Quantifying the contribution of sea lice from aquaculture to declining annual returns in a wild Atlantic salmon population. Aquacult Environ Interactions. (2017) 9:181–92. 10.3354/aei00223

[79] Grimnes A Jakobsen PJ. The physiological effects of salmon lice (*Lepeophtheirus salmonis*) infection on post smolts of Atlantic Salmon (*Salmo salar*). J Fish Biol. (1996) 48:1179–94. 10.1111/j.1095-8649.1996.tb01813.x10938722

[80] Brauner CJ Sackville M Gallagher Z Tang S Nendick L Farrell AP. Physiological consequences of the salmon louse (*Lepeophtheirus salmonis*) on juvenile pink salmon (*Oncorhynchus gorbuscha*): implications for wild salmon ecology and management, and for salmon aquaculture. Philos Trans Royal Soc B. (2012) 367:1770–9. 10.1098/rstb.2011.0423 22566682 PMC3350652

[81] Heuch PA Gettinby G Revie CW. Counting sea lice on Atlantic salmon farms- empirical and theoretical observations. Aquaculture. (2011) 320:149–53. 10.1016/j.aquaculture.2011.05.002

[82] Bui S Oppedal F Sievers M Dempster T. Behaviour in the toolbox to outsmart parasites and improve fish welfare in aquaculture. Rev Aquacult. (2019) 11:168–86. 10.1111/raq.12232

[83] Burka JF Hammell KL Horsberg TE Johnson GR Rainnie DJ Spear DJ. Drugs in salmonid aquaculture – a review. J Vet Pharmacol Ther. (1997) 20:333–49. 10.1046/j.1365-2885.1997.00094.x 9350253

[84] Vera LM Migaud H. Hydrogen peroxide treatment in Atlantic salmon induces stress and detoxification response in a daily manner. Chronobiol Int. (2016) 33:530–42. 10.3109/07420528.2015.1131164 27058450

[85] Overton K Dempster T Oppendal F Kristiansen TS Gismervik K Stien LH. Salmon lice treatments and salmon mortality in Norwegian aquaculture: a review. Rev Aquacult. (2019) 11:1398–417. 10.1111/raq.12299

[86] Aaen SM Helgesen KO Bakke MJ Kaur K Horsberg TE. Drug resistance in sea lice: a threat to salmonid aquaculture. Trends Parasitol. (2015) 31:72–81. 10.1016/j.pt.2014.12.006 25639521

[87] Denholm I Devine GJ Horsberg TE Sevatdal S Fallang A Nolan DV. Analysis and management of resistance to chemotherapeutants in salmon lice, *Lepeophtheirus salmonis* (Copepoda: Caligidae). Pest Manag Sci. (2002) 58:528–36. 10.1002/ps.482 12138619

[88] Ljungfeldt LER Espedal PG Nilsen F Skern-Mauritzen M Glover KA. A common-garden experiment to quantify evolutionary processes in copepods: the case of emamectin benzoate resistance in the parasitic sea louse *Lepeophtheirus salmonis*. BMC Evol Biol. (2014) 14:1–18. 10.1186/1471-2148-14-108 24885085 PMC4057923

[89] McNair CM. Ectoparasites of medical and veterinary importance: drug resistance and the need for alternative control methods. J Pharm Pharmacol. (2015) 67:351–63. 10.1111/jphp.12368 25644683

[90] Nilsson J Moltumyr L Madaro A Kristiansen TS Gåsnes SK Mejdell CM. Sudden exposure to warm water causes instant behavioural responses indicative of nociception or pain in Atlantic salmon. Vet Anim Sci. (2019) 8:100076. 10.1016/j.vas.2019.100076 32734093 PMC7386744

[91] Gismervik K Gåsnesa SK Gua J Stien LH Madarob A Nilsson J. Thermal injuries in Atlantic salmon in a pilot laboratory trial. Vet Anim Sci. (2019) 8:100081. 10.1016/j.vas.2019.100081 32734098 PMC7386709

[92] Småge SB Frisch K Brevik OJ Watanabe K Nylund A. First isolation, identification and characterisation of *Tenacibaculum maritimum* in Norway, isolated from diseased farmed sea lice cleaner fish *Cycolpterus lumpus* L. Aquaculture. (2016) 464:178–84. 10.1016/j.aquaculture.2016.06.030

[93] Treasurer JW. A review of potential pathogens of sea lice and the application of cleaner fish in biological control. Pest Manag Sci. (2002) 58:546–58. 10.1002/ps.509 12138621

[94] Brooker AJ Papadopoulou A Gutierrez C Rey S Davie A Migaud H. Sustainable production and use of cleaner fish for the biological control of sea lice: recent advances and current challenges. Vet Record. (2018) 183:383. 10.1136/vr.104966 30061113

[95] Powell A Treasurer JW Pooley CL Keay AJ Lloyd R Imsland AK. Use of lumpfish for sea-lice control in salmon farming: challenges and opportunities. Rev Aquacult. (2018) 10:683–702. 10.1111/raq.12194

[96] Halvorsen KT Larsen T Sørdalen TK Vøllestad LA Knutsen H Olsen EM. Impact of harvesting cleaner fish for salmonid aquaculture assessed from replicated coastal marine protected areas. Mar Biol Res. (2017) 13:359–69. 10.1080/17451000.2016.1262042

[97] Hvas M Folkedal O Imsland A Oppedal F. Metabolic rates, swimming capabilities, thermal niche and stress response of the lumpfish, *Cyclopterus lumpus*. Biol Open. (2018) 7:bio036079. 10.1242/bio.036079 30115616 PMC6176939

[98] Yuen JW Dempster T Oppedal F Hvas M. Physiological performance of ballan wrasse (*Labrus bergylta*) at different temperatures and its implication for cleaner fish usage in salmon aquaculture. Biol Control. (2019) 135:117–23. 10.1016/j.biocontrol.2019.05.007

[99] Stien LH StØrkersen KV Gåsnes SK. Analysis of mortality data from survey on cleaner fish. Rapport fra havforskningen. (2020) 6. Available online at: Available online at: https://www.hi.no/en/hi/nettrapporter/rapport-fra-havforskningen-2020-6 (accessed October 10, 2021).

[100] Skiftesvik AB Blom G Agnalt AL Durif CM Browman HI Bjelland RM. Wrasse (Labridae) as cleaner fish in salmonid aquaculture–the Hardangerfjord as a case study. Mar Biol Res. (2014) 10:289–300. 10.1080/17451000.2013.810760

[101] Nilsen A Viljugrein H Røsæg MV Colquhoun D. Rensefiskhelse–kartlegging av dødelighet og dødelighetsårsaker. Veterinærinstituttets Rapportserie. (2014) 12:1–844. Available online at: https://lusedata.no/wp-content/uploads/2014/09/Rapportserie-12-2014-Rensefiskhelse_kartlegging.pdf (accessed August 10, 2021)30277038

[102] Overton K Barrett LT Oppedal F Kristiansen TS Dempster T. Sea lice removal by cleaner fish in salmon aquaculture: a review of the evidence base. Aquacult Environ Interact. (2020) 12:31–44. 10.3354/aei00345

[103] Baeverfjord G Antony Jesu Prabhu P Fjelldal PG Albrektsen S Hatlen B Denstadli V. Mineral nutrition and bone health in salmonids. Rev Aquacult. (2019) 11:740–65. 10.1111/raq.1225520549801

[104] Gatlin DM Barrows FT Brown P Dabrowski K Gaylord TG Hardy RW. Expanding the utilization of sustainable plant products in aquafeeds: a review. Aquacult Res. (2007) 38:551–79. 10.1111/j.1365-2109.2007.01704.x

[105] Gasco L Gai F Maricchiolo G Genovese L Ragonese S Bottari T. Fishmeal Alternative Protein Sources. Feeds for the Aquaculture Sector: Current Situation and Alternative Sources. Cham: Springer (2018). p. 1–20. 10.1007/978-3-319-77941-6_1

[106] Cadillo-Benalcazar JJ Giampietro M Bukkens SG Strand R. Multi-scale integrated evaluation of the sustainability of large-scale use of alternative feeds in salmon aquaculture. J Clean Prod. (2020) 248:119210. 10.1016/j.jclepro.2019.119210

[107] Krogdahl A Bakke-McKellep AM Roed KH Baeverfjord G. Feeding Atlantic salmon *Salmo salar* L. soybean products: effects on disease resistance (furunculosis), and lysozyme and IgM levels in the intestinal mucosa. Aquacult Nutr. (2000) 6:77–84. 10.1046/j.1365-2095.2000.00129.x

[108] Booman M Forster I Vederas JC Groman DB Jones SR. Soybean meal-induced enteritis in Atlantic salmon (*Salmo salar*) and Chinook salmon (*Oncorhynchus tshawytscha*) but not in pink salmon (O. gorbuscha). Aquaculture. (2018) 483:238–43. 10.1016/j.aquaculture.2017.10.025

[109] Romarheim OH Øverland M Mydland LT Skrede A Landsverk T. Bacteria grown on natural gas prevent soybean meal-induced enteritis in Atlantic salmon. J Nutr. (2011) 141:124–30. 10.3945/jn.110.128900 21106922

[110] EFSA. Animal welfare aspects of husbandry systems for farmed Atlantic salmon. EFSA J. (2008) 736:1–31. 10.2903/j.efsa.2008.736 PMC10193664 37213864

[111] Burr GS Wolters WR Schrader KK Summerfelt ST. Impact of depuration of earthy-musty off-flavors on fillet quality of Atlantic salmon, *Salmo salar*, cultured in a recirculating aquaculture system. Aquacult Eng. (2012) 50:28–39. 10.1016/j.aquaeng.2012.03.002

[112] Saunders RL Muise BC Henderson EB. Mortality of salmonids cultured at low temperature in seawater. Aquaculture. (1975) 5:243–52. 10.1016/0044-8486(75)90002-2

[113] Fletcher GL Kao MH Dempson JB. Lethal freezing temperatures of Arctic char and other salmonids in the presence of ice. Aquaculture. (1988) 71:369–78. 10.1016/0044-8486(88)90206-2

[114] Quinones RA Fuentes M Montes RM Soto D Leon-Munoz J. Environmental issues in Chilean salmon farming: a review. Rev Aquacult. (2017) 11:375–402. 10.1111/raq.12337

[115] Wade NM Clark TD Maynard BT Atherton S Wilkinson RJ Smullen RP. Effects of an unprecedented summer heatwave on the growth performance, flesh colour and plasma biochemistry of marine cage-farmed Atlantic salmon (*Salmo salar*). J Therm Biol. (2019) 80:64–74. 10.1016/j.jtherbio.2018.12.021 30784489

[116] Brown C. Fish intelligence, sentience and ethics. Anim Cogn. (2015) 18:1–17. 10.1007/s10071-014-0761-0 24942105

[117] Stevenson RJ Mahmut M Rooney K. Individual differences in the interoceptive states of hunger, fullness and thirst. Appetite. (2015) 95:44–57. 10.1016/j.appet.2015.06.008 26119812

[118] Key B. Why fish do not feel pain. Animal Sentience. (2016) 1:1. 10.51291/2377-7478.1011

[119] Browman HI Cooke SJ Cowx IG Derbyshire SW Kasumyan A Key B. Welfare of aquatic animals: where things are, where they are going, and what it means for research, aquaculture, recreational angling, and commercial fishing. ICES J Mar Sci. (2019) 76:82–92. 10.1093/icesjms/fsy067

[120] Fry FEJ. The effect of environmental factors on the physiology of fish. In: WS Hoar, DJ Randall, editors, Fish Physiology. Vol. 6. Cambridge: Academic Press (1971). p. 1–98. 10.1016/S1546-5098(08)60146-6

[121] Hvas M Stien LH Oppedal F. The metabolic rate response to feed withdrawal in Atlantic salmon post-smolts. Aquaculture. (2020) 529:735690. 10.1016/j.aquaculture.2020.735690

[122] Cunjak RA Power G. The feeding and energetics of stream-resident trout in winter. J Fish Biol. (1987) 31:493–511. 10.1111/j.1095-8649.1987.tb05254.x

[123] Raven PA Uh M Sakhrani D Beckman BR Cooper K Pinter J. Endocrine effects of growth hormone overexpression in transgenic coho salmon. Gen Comp Endocrinol. (2008) 159:26–37. 10.1016/j.ygcen.2008.07.011 18713628

[124] Kim J-H Leggatt RA Chan M Volkoff H Devlin RH. Effects of chronic growth hormone overexpression on appetite-regulating brain gene expression in coho salmon. Mol Cell Endocrinol. (2015) 413:178–88. 10.1016/j.mce.2015.06.024 26123591

[125] White SL Volkoff H Devlin RH. Regulation of feeding behavior and food intake by appetite-regulating peptides in wild-type and growth hormone-transgenic coho salmon. Horm Behav. (2016) 84:18–28. 10.1016/j.yhbeh.2016.04.005 27149948

[126] Bar N. Physiological and hormonal changes during prolonged starvation in fish. Can J Fisheries Aquatic Sci. (2014) 71:1447–58. 10.1139/cjfas-2013-0175

[127] FAWC. Opinion on the Welfare of Farmed Fish. Farm Animal Welfare Council. (2014). Available online at: https://assets.publishing.service.gov.uk/government/uploads/system/uploads/attachment_data/file/319323/Opinion_on_the_welfare_of_farmed_fish.pdf (accessed August 12, 2021).

[128] Cañon Cañon Jones HA Noble C Damsgård B Pearce GP. Evaluating the effects of a short-term feed restriction period on the behavior and welfare of Atlantic salmon, *Salmo salar*, parr using social network analysis and fin damage. J World Aquac Soc. (2017) 48:35–45. 10.1111/jwas.12322

[129] EFSA. Species-specific welfare aspects of the main systems of stunning and killing of farmed fish: rainbow trout. EFSA J. (2009) 1013:1–55. 10.2903/j.efsa.2009.1012

[130] Clarke A Johnston NM. Scaling of metabolic rate with body mass and temperature in teleost fish. J Anim Ecol. (1999) 68:893–905. 10.1046/j.1365-2656.1999.00337.x

[131] Roh H Park J Kim A Kim N Lee Y Kim BS. Overfeeding-induced obesity could cause potential immuno-physiological disorders in rainbow trout (*Oncorhynchus mykiss*). Animals. (2020) 10:1499. 10.3390/ani10091499 32854279 PMC7552159

[132] Newberry RC. Environmental enrichment: increasing the biological relevance of captive environments. Appl Anim Behav Sci. (1995) 44:229–43. 10.1016/0168-1591(95)00616-Z

[133] Näslund J Johnsson JI. Environmental enrichment for fish in captive environments: effects of physical structures and substrates. Fish Fisheries. (2016) 17:1–30. 10.1111/faf.12088

[134] Fife-Cook I Franks B. Positive welfare for fishes: rationale and areas for future study. Fishes. (2019) 4:31. 10.3390/fishes4020031

[135] Bosakowski T Wagner EJ. Experimental use of cobble substrates in concrete raceways for improving fin condition of cutthroat (*Oncorhynchus clarki*) and rainbow trout (*O.mykiss*). Aquaculture. (1995) 130:159–65. 10.1016/0044-8486(94)00223-B

[136] Wagner EJ Intelmann SS Routledge MD. The effects of fry rearing density on hatchery performance, fin condition, and agonistic behavior of rainbow trout *Oncorhynchus mykiss* fry. J World Aquac Soc. (1996) 27:264–74. 10.1111/j.1749-7345.1996.tb00608.x

[137] Arndt RE Routledge MD Wagner EJ Mellenthin RF. Influence of raceway substrate and design on fin erosion and hatchery performance of rainbow trout. North Am J Aquacult. (2001) 63:312–20. 10.1577/1548-8454(2001)063<0312:IORSAD>2.0.CO;2

[138] Mikheev VN Adams CE Huntingford FA Thorpe JE. Behavioural responses of benthic and pelagic Arctic charr to substratum heterogeneity. J Fish Biol. (1996) 49:494–500. 10.1111/j.1095-8649.1996.tb00044.x

[139] Gaffney LP Franks B Weary DM von Keyserlingk MA. Coho salmon (*Oncorhynchus kisutch*) prefer and are less aggressive in darker environments. PLoS ONE. (2016) 11:e0151325. 10.1371/journal.pone.0151325 27028731 PMC4814047

[140] Pickering AD Griffiths R Pottinger TG. A comparison of the effects of overhead cover on the growth survival and haematology of juvenile Atlantic salmon, *Salmo salar* L, brown trout, *Salmo trutta* L, and rainbow trout, *Salmo gairdneri* Richardson. Aquaculture. (1987) 66:109–24. 10.1016/0044-8486(87)90226-2

[141] Räihä V Sundberg LR Ashrafi R Hyvärinen P Karvonen A. Rearing background and exposure environment together explain higher survival of aquaculture fish during a bacterial outbreak. J Appl Ecol. (2019) 56:1741–50. 10.1111/1365-2664.13393

[142] Baynes SM Howell BR. Observations on the growth survival and disease resistance of juvenile common sole, *Solea solea* (L), fed *Mytilus edulis* L. Aquacult Res. (1993) 24:95–100. 10.1111/j.1365-2109.1993.tb00831.x

[143] Tuckey LM Smith TI. Effects of photoperiod and substrate on larval development and substrate preference of juvenile southern flounder, *Paralichthys lethostigma*. J Appl Aquacult. (2001) 11:1–20. 10.1300/J028v11n01_02

[144] Taranger GL Haux C Hansen T Stefansson SO Björnsson BT Walther BT. Mechanisms underlying photoperiodic effects on age at sexual maturity in Atlantic salmon, *Salmo salar*. Aquaculture. (1999) 177:47–60. 10.1016/S0044-8486(99)00068-X

[145] Handeland SO Stefansson SO. Photoperiod control and influence of body size on off-season parr–smolt transformation and post-smolt growth. Aquaculture. (2001) 192:291–3. 10.1016/S0044-8486(00)00457-9

[146] Fjelldal PG Lock EJ Grotmol S Totland GK Nordgarden U Flik G. Impact of smolt production strategy on vertebral growth and mineralisation during smoltification and the early seawater phase in Atlantic salmon (*Salmo salar.* L) Aquaculture. (2006) 261:715–28. 10.1016/j.aquaculture.2006.08.008

[147] Ebbesson LO Ebbesson SO Nilsen TO Stefansson SO Holmqvist B. Exposure to continuous light disrupts retinal innervation of the preoptic nucleus during parr–smolt transformation in Atlantic salmon. Aquaculture. (2007) 273:345–9. 10.1016/j.aquaculture.2007.10.016

[148] Mork OI Gulbrandsen J. Vertical activity of four salmonid species in response to changes between darkness and two intensities of light. Aquaculture. (1994) 127:317–28. 10.1016/0044-8486(94)90234-8

[149] Clark CW Levy DA. Diel vertical migrations by juvenile sockeye salmon and the antipredation window. Am Nat. (1988) 131:271–90. 10.1086/284789

[150] Folkedal O Torgersen T Nilsson J Oppedal F. Habituation rate and capacity of Atlantic salmon (*Salmo salar*) parr to sudden transitions from darkness to light. Aquaculture. (2010) 307:170–2. 10.1016/j.aquaculture.2010.06.001

[151] Handeland SO Imsland AK Ebbesson LO Nilsen TO Hosfeld CD Baeverfjord G. Low light intensity can reduce Atlantic salmon smolt quality. Aquaculture. (2013) 384:19–24. 10.1016/j.aquaculture.2012.12.016

[152] CVMA. Euthanasia- Position Statement. Canadian Veterinarian Medical Association (2014). Available online at: https://www.canadianveterinarians.net/documents/euthanasia (accessed August 3, 2021).

[153] AVMA. AVMA Guidelines for the Humane Slaughter of Animals: 2016 Edition. American Veterinary Medical Association (2016). Available online at: https://www.avma.org/sites/default/files/resources/Humane-Slaughter-Guidelines.pdf (accessed August 2, 2021).

[154] OIE. Chapter 7.3-Welfare Aspects of Stunning and Killing of Farmed Fish for Human Consumption. World Organisation for Animal Health (2021). Available online at: https://www.oie.int/en/what-we-do/standards/codes-and-manuals/aquatic-code-online-access/?id=169andL=1andhtmfile=chapitre_welfare_stunning_killing.htm (accessed October 12, 2021).

[155] Barthel BL Cooke SJ Suski CD Philipp DP. Effects of landing net mesh type on injury and mortality in a freshwater recreational fishery. Fish Res. (2003) 63:275–82. 10.1016/S0165-7836(03)00059-6

[156] Lines JA Spence J. Safeguarding the welfare of farmed fish at Harvest. Fish Physiol Biochem. (2011) 38:153–62. 10.1007/s10695-011-9561-5 21989953

[157] Erikson U Gansel L Frank K Svendsen E Digre H. Crowding of Atlantic salmon in net-pen before slaughter. Aquaculture. (2016) 465:395–400. 10.1016/j.aquaculture.2016.09.018

[158] Iversen M Finstad B McKinley RS Eliassen RA Carlsen KT Evjen T. Stress responses in Atlantic salmon (*Salmo salar* L.) smolts during commercial well boat transports and effects on survival after transfer to sea. Aquaculture. (2005) 243:373–82. 10.1016/j.aquaculture.2004.10.019

[159] Morzel M Sohier D Van de Vis H. Evaluation of slaughtering methods for turbot with respect to animal welfare and flesh quality. J Sci Food Agric. (2003) 83:19–28. 10.1002/jsfa.125325855820

[160] Erikson U Sigholt T Seland A. Handling stress and water quality during live transportation and slaughter of Atlantic salmon (*Salmo salar*). Aquaculture. (1997) 149:243–52. 10.1016/S0044-8486(96)01453-6

[161] Robb DHF Kestin SC Warriss PD. Muscle activity at slaughter. I Changes in flesh colour and gaping in rainbow trout. Aquaculture. (2000) 182:261–9. 10.1016/S0044-8486(99)00273-2

[162] Robb DHF Wotton SB McKinstry JL Sorensen NK Kestin SC. Commercial slaughter methods used on Atlantis salmon: determination of the onset of brain failure by electroencephalography. Vet Record. (2000) 147:298–303. 10.1136/vr.147.11.298 11037730

[163] Lines JA Robb DH Kestin SC Crook SC Benson T. Electric stunning: a humane slaughter method for trout. Aquacult Eng. (2003) 28:141–54. 10.1016/S0144-8609(03)00021-932539725

[164] Roth B Imsland A Gunnarsson S Foss A Schelvis-Smit R. Slaughter quality and rigor contraction in farmed turbot (*Scophthalmus maximus*); a comparison between different stunning methods. Aquaculture. (2007) 272:754–61. 10.1016/j.aquaculture.2007.09.012

[165] Van de Vis H Kestin S Robb D Oehlenschlager J Lambooij B Munkner W. Is humane slaughter of fish possible for industry? Aquac Res. (2003) 34:211–20. 10.1046/j.1365-2109.2003.00804.x

[166] SANTE. Welfare of Farmed Fish: Common Practices During Transport and at Slaughter. European Commission Directorate Health and Food Safety (2017). Available online at: http://publications.europa.eu/resource/cellar/facddd32-cda6-11e7-a5d5-01aa75ed71a1.0001.01/DOC_1 (accessed November 1, 2021).

[167] Braithwaite VA Ebbesson LOE. Pain and stress responses in farmed fish. Revue Scientifique et Technique. (2014) 33:245–53. 10.20506/rst.33.1.2285 25000797

[168] Lines J Kestin S. Electrical stunning of fish: the relationship between the electric field strength and water conductivity. Aquaculture. (2004) 241:219–34. 10.1016/j.aquaculture.2004.07.023

[169] Nieuwenhuys R. The forebrain of actinopterygians revisited. Brain Behav Evol. (2009) 73:229–52. 10.1159/000225622 19546532

[170] Kestin SC van de Vis JW Robb DHF. Protocol for assessing brain function in fish and the effectiveness of methods used to stun and kill them. Vet Record. (2002) 150:302–7. 10.1136/vr.150.10.302 11915866

[171] Robb DHF Roth B. Brain activity of Atlantic salmon (*Salmo salar*) following electrical stunning using various field strengths and pulse durations. Aquaculture. (2003) 216:363–9. 10.1016/S0044-8486(02)00494-5

[172] Bowman J Hjelmstedt P Gräns A. Non-invasive recording of brain function in rainbow trout: evaluations of the effects of MS-222 anaesthesia induction. Aquac Res. (2019) 50:3420–8. 10.1111/are.14300

[173] Bowman J van Nuland N Hjelmstedt P Berg C Gräns A. Evaluation of the reliability of indicators of consciousness during CO_2_ stunning of rainbow trout and the effects of temperature. Aquac Res. (2020) 51:5194–202. 10.1111/are.14857

[174] Van de Vis H Abbink W Lambooij B Bracke M. Stunning and killing of farmed fish: how to put it into practice? In: Encyclopedia of Meat Sciences. 2nd ed. Cambridge. MA: Academic Press (2014). p. 421–6. 10.1016/B978-0-12-384731-7.00199-9

[175] Roque A Gras N Rey-Planellas S Fatsini E Pallisera J Duncan N. The feasibility of using gas mixture to stun seabream (*Sparus aurata*) before slaughtering in aquaculture production. Aquaculture. (2021) 545:737168. 10.1016/j.aquaculture.2021.737168

[176] Kestin SC Wotton SB Gregory NG. Effect of slaughter by removal from water on visual evoked activity in the brain and reflex movement of rainbow trout (*Oncorhynchus mykiss*). Vet Record. (1991) 128:443–6. 10.1136/vr.128.19.443 1858271

[177] Peters MDJ Godfrey C McInerney P Munn Z Tricco AC Khalil H. Chapter 11: scoping reviews. In: E Aromataris, Z Munn, editors, JBI Manual for Evidence Synthesis. JBI (2020). 10.46658/JBIMES-20-12 (accessed August 10, 2021).

[178] Manfrin A Messori S Arcangeli G. Strengthening fish welfare research through a gap analysis study. Report for SCAR FISH and SCAR CWG. Anim Health Welfare Res. (2018). Available online at: https://scar-europe.org/images/FISH/Documents/Report_CWG-AHW_CASA_FISH-welfare.pdf (accessed August 10, 2021).

[179] Macaulay G Bui S Oppedal F Dempster T. Challenges and benefits of applying fish behaviour to improve production and welfare in industrial aquaculture. Rev Aquacult. (2021) 13:934–48. 10.1111/raq.12505

[180] Hvas M Folkedal O Oppedal F. Fish welfare in offshore salmon aquaculture. Rev Aquacult. (2021) 13:836–52. 10.1111/raq.12501

[181] McArthur A Klugarova J Yan H Florescu S. Chapter 4: systematic reviews of text and opinion. In: E Aromataris, Z Munn, editors, JBI Manual for Evidence Synthesis. JBI (2020). 10.46658/JBIMES-20-05 (accessed August 10, 2021).

[182] Jhangiani RS Chiang IA Price PC. Research Methods in Psychology, 2nd Canadian ed. Victoria, BC: BC Campus. (2015).

[183] Pavlidis M Digka N Theodoridi A Campo A Barsakis K Skouradakis G. Husbandry of zebrafish, *Danio rerio*, and the cortisol stress response. Zebrafish. (2013) 10:524–31. 10.1089/zeb.2012.0819 23886279

[184] Hoyle I Oidtmann B Ellis T Turnbull J North B Nikolaidis J. A validated macroscopic key to assess fin damage in farmed rainbow trout (*Oncorhynchus mykiss*). Aquaculture. (2007) 270:142–8. 10.1016/j.aquaculture.2007.03.037

[185] Hartman KJ Margraf FJ Hafs AW Cox MK. Bioelectrical impedance analysis: a new tool for assessing fish condition. Fisheries. (2015) 40:590–600. 10.1080/03632415.2015.1106943

[186] Fazio F. Fish hematology analysis as an important tool of aquaculture: a review. Aquaculture. (2019) 500:237–42. 10.1016/j.aquaculture.2018.10.030

[187] Sopinka NM Donaldson MR O'Connor CM Suski CD Cooke SJ. Stress indicators in fish. In: Fish Physiology. Vol. 35. Cambridge, MA: Academic Press (2016). p. 405–62. 10.1016/B978-0-12-802728-8.00011-4

[188] Cone RS. The need to reconsider the use of condition indices in fishery science. Trans Am Fish Soc. (1989) 118:510–4.

[189] Morton A Routledge RD. Fulton's condition factor: is it a valid measure of sea lice impact on juvenile salmon? North Am J Fisheries Manag. (2006) 26:56–62. 10.1577/M05-068.1

[190] Duncan IJ. Measuring preferences and the strength of preferences. Poult Sci. (1992) 71:658–63. 10.3382/ps.0710658 1594518

[191] Schroeder P Jones S Young IS Sneddon LU. What do zebrafish want? Impact of social grouping, dominance and gender on preference for enrichment. Lab Anim. (2014) 48:328–37. 10.1177/0023677214538239 24939904

[192] Dawkins MS. Battery hens name their price: consumer demand theory and the measurement of ethological “needs”. Anim Behav. (1983) 31:1195–205. 10.1016/S0003-3472(83)80026-8

[193] Mason GJ Cooper J Clarebrough C. Frustrations of fur-farmed mink. Nature. (2001) 410:35–6. 10.1038/35065157 11242031

[194] Blanchette I Richards A. The influence of affect on higher level cognition: a review of research on interpretation, judgement, decision making and reasoning. Cogn Emot. (2010) 24:561–95. 10.1080/02699930903132496

[195] Harding EJ Paul ES Mendl M. Cognitive bias and affective state. Nature. (2004) 427:312–2. 10.1038/427312a 14737158

[196] Resasco A MacLellan A Ayala MA Kitchenham L Edwards AM Lam S. Cancer blues? A promising judgment bias task indicates pessimism in nude mice with tumors. Physiol Behav. (2021) 2021:113465. 10.1016/j.physbeh.2021.113465 34029586

[197] Espigares F Abad-Tortosa D Varela SAM Ferreira MG Oliveira RF. Short telomeres drive pessimistic judgement bias in zebrafish. Biol Lett. (2021) 17:20200745. 10.1098/rsbl.2020.0745 33726560 PMC8086985

[198] Cunjak RA Prowse TD Parrish DL. Atlantic salmon (*Salmo salar*) in winter: “the season of parr discontent?” *Can J Fisheries Aquatic Sci*. (1998) 55:161–80. 10.1139/d98-008

[199] Fleming IA. Reproductive strategies of Atlantic salmon: ecology and evolution. Rev Fish Biol Fish. (1996) 6:379–416. 10.1007/BF00164323

[200] Durán E Ocana FM Martín-Monzón I Rodríguez F Salas C. Cerebellum and spatial cognition in goldfish. Behav Brain Res. (2014) 259:1–8. 10.1016/j.bbr.2013.10.039 24184084

[201] Rodríguez-Expósito B Gómez A Martín-Monzón I Reiriz M Rodríguez F Salas C. Goldfish hippocampal pallium is essential to associate temporally discontiguous events. Neurobiol Learn Mem. (2017) 139:128–34. 10.1016/j.nlm.2017.01.002 28065713

[202] Schjolden J Stoskhus A Winberg S. Does individual variation in stress responses and agonistic behavior reflect divergent stress coping strategies in juvenile rainbow trout? Physiol Biochem Zool. (2005) 78:715–23. 10.1086/432153 16075393

